# Time to Renal Disease and End-Stage Renal Disease in PROFILE: A Multiethnic Lupus Cohort

**DOI:** 10.1371/journal.pmed.0030396

**Published:** 2006-10-31

**Authors:** Graciela S Alarcón, Gerald McGwin, Michelle Petri, Rosalind Ramsey-Goldman, Barri J Fessler, Luis M Vilá, Jeffrey C Edberg, John D Reveille, Robert P Kimberly, for the PROFILE Study Group

**Affiliations:** 1 Division of Clinical Immunology and Rheumatology, Department of Medicine, School of Medicine, University of Alabama at Birmingham, Birmingham, Alabama, United States of America; 2 Section of Trauma, Burns, and Critical Care, Department of Surgery, School of Medicine, University of Alabama at Birmingham, Birmingham, Alabama, United States of America; 3 Division of Rheumatology, School of Medicine, Johns Hopkins University, Baltimore, Maryland, United States of America; 4 Division of Rheumatology, Northwestern University School of Medicine, Chicago, Illinois, United States of America; 5 Division of Rheumatology, Department of Medicine, University of Puerto Rico Medical Sciences Campus, San Juan, Puerto Rico; 6 Division of Rheumatology, Department of Medicine, University of Texas Health Science Center at Houston, Houston, Texas, United States of America; Lund University Hospital, Sweden

## Abstract

**Background:**

Renal involvement is a serious manifestation of systemic lupus erythematosus (SLE); it may portend a poor prognosis as it may lead to end-stage renal disease (ESRD). The purpose of this study was to determine the factors predicting the development of renal involvement and its progression to ESRD in a multi-ethnic SLE cohort (PROFILE).

**Methods and Findings:**

PROFILE includes SLE patients from five different United States institutions. We examined at baseline the socioeconomic–demographic, clinical, and genetic variables associated with the development of renal involvement and its progression to ESRD by univariable and multivariable Cox proportional hazards regression analyses. Analyses of onset of renal involvement included only patients with renal involvement after SLE diagnosis (*n =* 229). Analyses of ESRD included all patients, regardless of whether renal involvement occurred before, at, or after SLE diagnosis (34 of 438 patients). In addition, we performed a multivariable logistic regression analysis of the variables associated with the development of renal involvement at any time during the course of SLE.

In the time-dependent multivariable analysis, patients developing renal involvement were more likely to have more American College of Rheumatology criteria for SLE, and to be younger, hypertensive, and of African-American or Hispanic (from Texas) ethnicity. Alternative regression models were consistent with these results. In addition to greater accrued disease damage (renal damage excluded), younger age, and Hispanic ethnicity (from Texas), homozygosity for the valine allele of FcγRIIIa (FCGR3A*GG) was a significant predictor of ESRD. Results from the multivariable logistic regression model that included all cases of renal involvement were consistent with those from the Cox model.

**Conclusions:**

Fcγ receptor genotype is a risk factor for progression of renal disease to ESRD. Since the frequency distribution of *FCGR3A* alleles does not vary significantly among the ethnic groups studied, the additional factors underlying the ethnic disparities in renal disease progression remain to be elucidated.

## Abstract 2

Fcγ receptor genotype is a risk factor for progression of renal disease to ESRD but does not explain the ethnic disparities in renal disease progression.

## Editors' Summary

**Background.:**

Systemic lupus erythematosis (SLE, commonly known as “lupus”) is an illness of many manifestations that appear to result from the immune system attacking components of the body's own cells. One of the unfortunate effects of SLE is kidney damage, which can, in a minority of patients, progress to kidney failure (formally called “end-stage renal disease,” or ESRD). Compared to White Americans, other ethnic groups tend to develop renal complications of lupus more often and with worse outcomes.

**Why Was This Study Done?:**

It is unclear why some people with lupus develop kidney problems. The purpose of this US-based study was to confirm the factors that increase the risk of kidney damage and kidney failure, particularly in racial and ethnic minority patients, and to determine which of these factors accelerate the pace of kidney disease. Knowing these risk factors could allow the development and targeting of interventions, such as screening tests and preventive treatments, to prevent long-term loss of kidney function in patients with lupus.

**What Did the Researchers Do and Find?:**

The researchers measured a number of factors in a multi-ethnic group of 1,008 patients with lupus, almost half of whom had some degree of kidney involvement. They found that those who developed kidney damage after being diagnosed with lupus tended to be younger, to have had lupus for a longer time, and to have experienced more effects of lupus in general than those who did not have kidney involvement. Those who developed kidney problems were also more likely to have been unemployed, to have had fewer years of formal education, and to have had high blood pressure before developing kidney involvement. African-American and Texan Hispanic individuals with lupus were more likely to develop kidney involvement, and tended to develop it more rapidly, than White Americans or Puerto Rican Hispanic ethnicity. Actual kidney failure (ESRD requiring dialysis or kidney transplantation) was more likely to occur among Texan Hispanics with kidney involvement than in the other ethnic groups. Diabetes and high blood pressure were not found to predict ESRD, but people with a particular variant of a protein that helps antibodies bind to cells (know as Fc-gamma receptor IIIa, or FcγRIIIa) were found to be more likely to develop ESRD, and to develop it more quickly.

**What Do These Findings Mean?:**

These results suggest that the emergence and progression of kidney disease in patients with lupus depends on medical, genetic, and socioeconomic factors. Because no single test or intervention can be expected to address all of these factors, those treating patients with lupus must remain aware of the complexity of their patients lives at a variety of levels. In particular, ethnic disparities in the risk of serious kidney disease remain to be addressed.

**Additional Information.:**

Please access these Web sites via the online version of this summary at http://dx.doi.org/10.1371/journal.pmed.0030396.

- MedlinePlus page on lupus
- Lupus Foundation of America
- American College of Rheumatology pages on lupus
- Wikipedia entry on lupus (note: Wikipedia is a free Internet encyclopedia that anyone can edit)

## Introduction

Systemic lupus erythematosus (SLE) is a prototypic immune complex disease characterized by a variable course and outcome. Renal involvement, one of the most serious manifestations, is clinically heterogeneous. Even among patients with the most severe histological forms of renal involvement, not all patients develop end-stage renal disease (ESRD) [1–3]. Compared to White Americans (Caucasians), other ethnic groups around the world tend to develop renal disease more frequently and to have worse outcomes [4–12].

Familial aggregation of SLE suggests a genetic component to disease susceptibility. Numerous studies have now found genetic associations with individual candidate genes [13–16]. Amongst the most robust candidate genes studied to date, those encoding HLA-DR, low-affinity Fc receptors for IgG *(FCGR),* (C4) complement, IRF5, mannose-binding lectin, CTLA4, and programmed cell death-1 have shown consistent association with SLE. Other candidate genes include certain cytokines and the Fas/FasL system. Of particular interest to the development of renal disease in patients with SLE is the reported association of renal disease with the low binding allele of *FCGR3A* (the FCGR3A*T allele), a finding supported by a recent meta-analysis [17,18]. Both global SLE phenotype and renal disease in patients with IgG2 anti-C1q autoantibodies have been associated with alleles of *FCGR2A* [19]. Recently, variation at the *FCGR3B* locus has also been related to renal disease in patients with SLE [20], making these three *FCGR* genes strong candidates for association with end organ damage and ESRD.

We have previously reported on a multi-ethnic, multi-center cohort of SLE patients, named PROFILE [21]. This cohort was constituted in 1998 by combining the existing cohorts at Northwestern University, Johns Hopkins University, the University of Alabama at Birmingham, the University of Texas Health Science Center at Houston, and the University of Puerto Rico Medical Sciences Campus (UPR) [22]. The underlying hypothesis was that the patients' genetic profile might allow for the prediction of their disease phenotype, hence the name PROFILE. We perform time-dependent analyses to determine the pace at which renal involvement and ESRD occurred in this SLE cohort. These data provide insight into the pathogenesis of these events and shed light on potential preventive strategies.

## Methods

Institutional review board approval to constitute this cohort and to follow these patients over time was obtained at each participating institution in accordance with the Declaration of Helsinki.

As previously described [21], PROFILE is a multi-institutional cohort of SLE patients. PROFILE patients are those from the individual cohorts who meet the American College of Rheumatology (ACR) revised and updated criteria [23,24], are 16 y of age or older, and have disease duration ≤10 y at the time they enter this cohort. They are also of defined ethnicity (Hispanic of Mexican ancestry [residing and enrolled in Texas, hence Texan Hispanics], Hispanic of Puerto Rican ancestry [residing and enrolled in Puerto Rico, hence Puerto Rican Hispanics], African-American, and White Americans), having reported all four grandparents to be of the same ethnic background. The patients are geographically distributed as follows: 132 patients from Northwestern University, 373 from Johns Hopkins University, 240 from University of Alabama at Birmingham, 171 from University of Texas Health Science Center at Houston, and 92 from UPR. The PROFILE database consists of those variables common to the individual cohorts that were identified after carefully mapping the different cohorts' databases [21]. The variables for the current analyses include both socioeconomic–demographic elements (age, gender, education, employment, marital status, and smoking) and clinical elements (disease duration, the number of ACR criteria at entry into the cohort [renal criterion excluded], renal involvement, ESRD, damage as assessed by the Systemic Lupus International Collaborating Clinics Damage Index [SDI], and medication intake [cyclophosphamide, azathioprine, mycophenolate mofetil, and glucocorticoids] [25,26]). For the purpose of these analyses, however, the SDI renal domain items were excluded. Comorbidities (diabetes [intake of oral hypoglycemic agents or insulin] and hypertension [recording of three abnormal readings or use of antihypertensive medications]) and the use of cyclophosphamide were also included.

Time of diagnosis was defined as the date at which a patient met four ACR criteria; therefore, renal involvement could have occurred prior to SLE diagnosis, at diagnosis, or after diagnosis. Disease duration was defined as the time between diagnosis and enrollment into the PROFILE cohort. Renal involvement was defined as present at the time patients met the ACR renal disorder criterion [23] or had biopsy-proven World Health Organization Class II–V lupus nephritis (for those in whom these data were available). ESRD was defined as the need for dialysis or transplantation.

From the genetic domain, selected HLA-DRB1 (HLA-DRB1*01, HLA-DRB1*0301, and HLA-DRB1*08), *HLA-DQB1* (HLA-DQB1*0201 and HLA-DQB1*0602), *HLA-DQA1* (HLA-DQA1*0501), *FCGR2A*, *FCGR3A*, and *FCGR3B* alleles were included. Genomic DNA was extracted using the PureGene kit (Gentra Systems, Minneapolis, Minnesota, United States) following the manufacturer's recommendations. *HLA-DRB1*, *HLA-DQB1,* and *HLA-DQA1* were genotyped as we have previously described [22]. *FCGR2A, FCGR3A,* and *FCGR3B* were genotyped as previously described and by Pyrosequencing (Biotage, Charlottesville, Virginia, United States) using gene-specific primers [27]. The distributions of selected *HLA-DR, HLA-DQ,* and *FCGR* alleles by ethnic group are included in Table S1.

**Figure 1:**
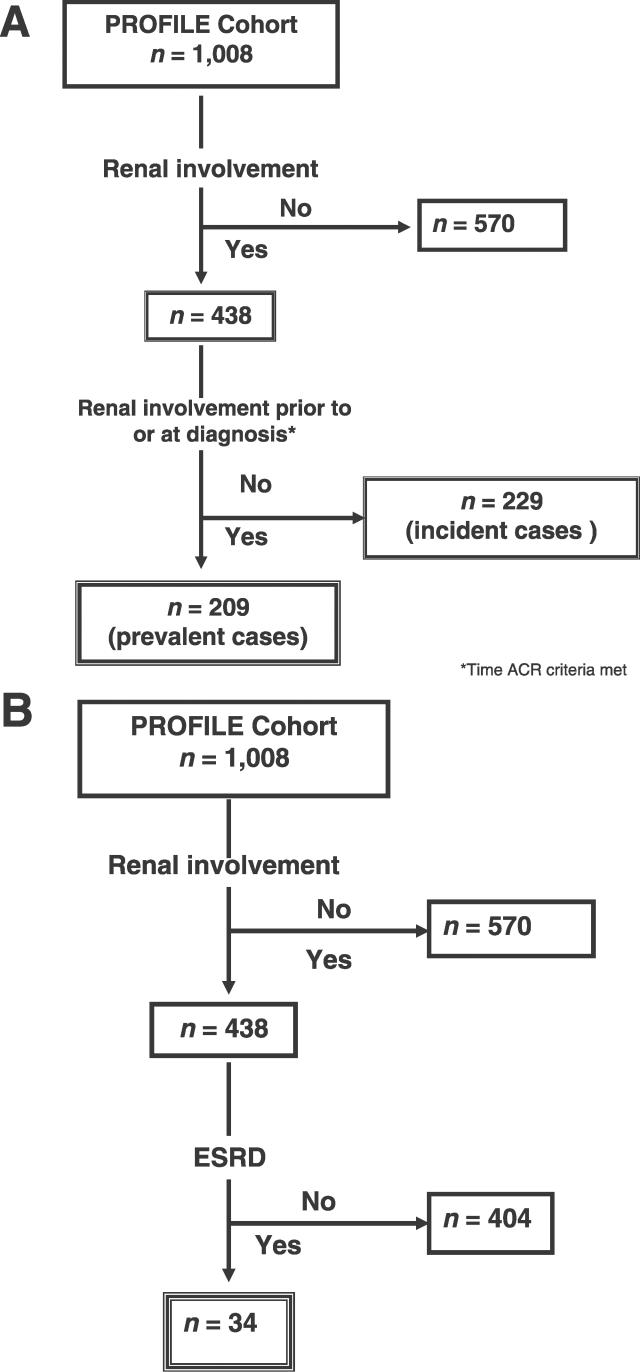
Flow Diagram of PROFILE Cohort Patients Included in Analyses (A) Renal involvement (double-lined boxes). (B) ESRD (triple-lined box).

The common variables from the individual databases were pooled to constitute one single database [21]. Descriptive statistical tests (Chi-square for proportions and Student's tests for means) were used to compare variables from the different domains for incident cases of renal involvement and for incident and prevalent cases taken together versus non-cases (prevalent cases, defined as those occurring prior to SLE diagnosis). Descriptive comparisons were also made between all patients whose renal involvement (prevalent and incident cases) had evolved into ESRD and those whose renal involvement had not. The effects of *FCGR3A* genotypes on the occurrence of ESRD were examined by Kaplan-Meier survival analysis. Variables with *p* < 0.10 in the univariable analyses and those felt to be clinically relevant regardless of their level of significance (e.g., hypertension) were entered into Cox proportional hazards regression models. The dependent variable was time to renal involvement in one model and time to ESRD in the other. In the case of renal involvement only those patients who developed it after entering the cohort were included in the model. Gender, age, and ethnicity were entered in both models. Occupation was excluded in the ESRD model since changes in patients' occupation status may result from ESRD rather than predict its occurrence. In two alternative models for renal involvement, treatment center rather than ethnicity was entered in one, and both treatment center and ethnicity were examined in the other; UPR patients were excluded in this analysis since all UPR patients have the same ethnicity. Finally, in another model all patients who developed renal involvement regardless of when it occurred were included; given that many of the independent variables were ascertained at baseline and not at diagnosis, a multivariable logistic regression rather than a Cox multivariable analysis of time to the event was performed. Histopathological data were not included in the ESRD regression given that this information was not available in nearly 45% of all patients. All analyses were done using SAS software, version 8.1 (SAS Institute, Cary, North Carolina, United States). Figure 1 depicts the flow of PROFILE patients into the time-dependent analyses described.

## Results

### Renal Involvement

At the time of analysis, 1,008 patients constituted the PROFILE cohort, 438 of whom had developed renal involvement (Figure 1A). Of these 438 patients, 209 had developed renal involvement prior to or at diagnosis (prevalent cases). At baseline, the 229 incident cases (those who developed renal involvement after SLE diagnosis) were younger (36.1 versus 39.7 y, *p* < 0.001) and more likely to be unemployed (48.9% versus 35.6%, *p* < 0.001), to have had fewer years of formal education (13.0 versus 13.9 y, *p =* 0.001), to have a greater number of ACR criteria (excluding the renal criterion) (7.0 versus 5.5, *p* < 0.001), to have had a longer disease duration (4.8 versus 2.7 y, *p* < 0.001), and to be hypertensive (45.7% versus 26.3%, *p* < 0.001) compared to the ones who had not developed renal involvement. African-Americans (105 of 237 patients, 44%) and Texan Hispanics (38 of 83 patients, 45%) were more likely to have developed renal involvement than the White Americans (73 of 392 patients, 18%) and Puerto Rican Hispanics (eight of 82 patients, 9%). These data are depicted in Table 1. The distribution of *HLA-DRB1, HLA-DQA1, HLA-DQB1,* and *FCGR* alleles were comparable for those patients who had developed and those who had not developed renal involvement (data not shown). Similar comparisons were carried out for all incident and prevalent cases together. These analyses were consistent with the data presented in Table 1 with the exception of smoking, which appeared to be somewhat less frequent among those who developed versus those who did not develop renal involvement (data not shown). In terms of the *HLA-DR, HLA-DQ,* and *FCGR* alleles, the data were also comparable between those with and without renal involvement with the exception of HLA-DRB1*1503, which associates with African-American ethnicity and which was found to be more frequent among those with renal involvement (13.5%) than among those without it (9.9%) (*p* < 0.001).

**Table 1:**
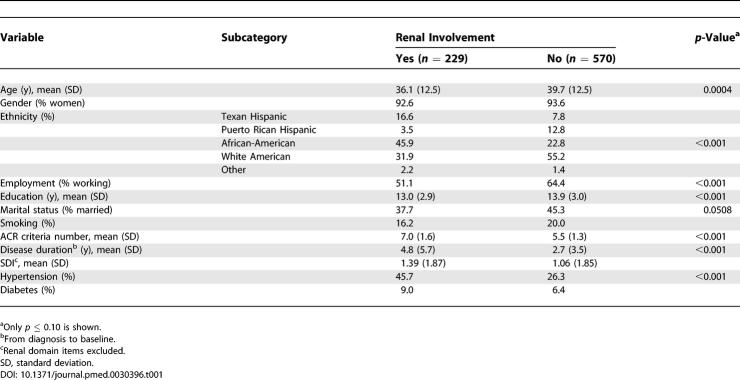
Baseline Socioeconomic–Demographic and Clinical Features of PROFILE Patients as a Function of Renal Involvement (Incident Cases)

### End-Stage Renal Disease

**Figure 2:**
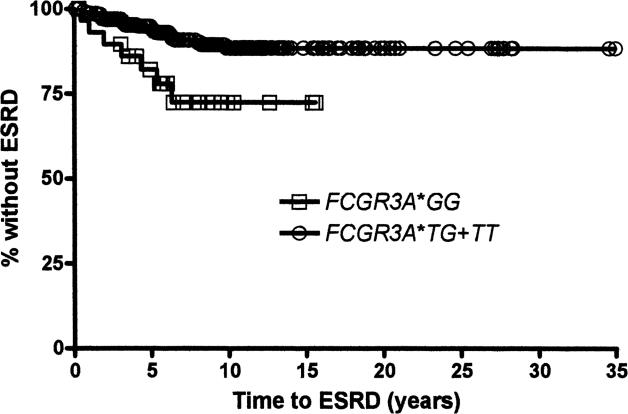
Kaplan-Meier Survival Curve for the Development of ESRD in Patients with FCGR3A*GG and FCGR3A*TT/GT

As shown in Figure 1B, 34 patients, from a total of 438 patients with renal involvement (incident and prevalent cases), had developed ESRD at the time of these analyses. Renal biopsies were not required for entry into the PROFILE database, but none of these patients had a lupus-scleroderma or other overlap syndrome to which ESRD could have been attributed. Very few of our patients received nonsteroidal anti-inflammatory drugs once renal involvement occurred; thus, nonsteroidal anti-inflammatory drug use as a principal contributing factor for the evolution into ESRD was considered highly unlikely. As shown in Table 2, patients whose condition evolved into ESRD were younger (31.8 versus 36.5 y, *p =* 0.0364) and more likely to be unemployed (75.8% versus 43.1%, *p* < 0.001), to have had fewer years of formal education (11.9 versus 13.2 y, *p =* 0.0218), to have had a shorter disease duration at entry into the cohort (1.0 versus 4.8 y, *p =* 0.003), and to have accrued more damage (excluding all renal domain items) (2.18 versus 1.43, *p =* 0.0280) at entry into the cohort. Within the different ethnic groups, ESRD was more likely to occur among Texan Hispanics affected with renal involvement (10 of 54 patients, 15%) than among patients in the other ethnic groups (24 of 374 patients, 6%, *p =* 0.0233). Hypertension, diabetes, and the use of cyclophosphamide, azathioprine, and mycophenolate mofetil occurred with comparable frequency in those patients who developed and those who did not develop ESRD. When these immunosuppressants were considered as a group, however, patients whose renal disease evolved into ESRD were more likely to have been treated with them than those whose disease did not, but the highest dose of glucocorticoids (as prednisone equivalent) was comparable in both groups. There was an overrepresentation of FCGR3A*GG among those patients who developed ESRD (21.9%) versus those who did not develop it (7.5%) (*p =* 0.0175), but the distribution of the other *FCGR2A* and *FCGR3B* alleles and of *HLA-DRB1, HLA-DQ1,* and *HLA-DQB1* alleles were comparable for those patients reaching and not reaching ESRD (data not shown). Figure 2 depicts the Kaplan-Meier survival curve of the genotypic effects of FCGR3A*GG versus FCGR3A*TT/GT on ESRD development.

**Table 2:**
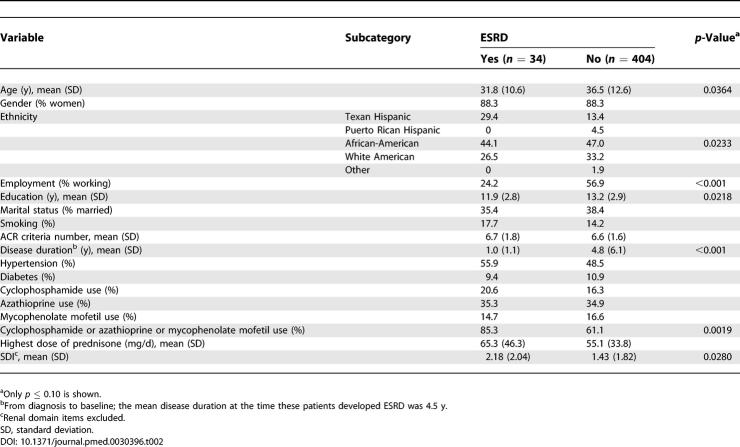
Baseline Socioeconomic–Demographic and Clinical Features of PROFILE Patients with Renal Involvement (Incident and Prevalent Cases) as a Function of the Occurrence of ESRD

### Multivariable Analyses: Time to Renal Involvement

The results of the Cox proportional hazard regression are shown in Table 3. Variables independently associated with time to the occurrence of renal involvement were younger age (hazard ratio [HR] = 0.975; 95% confidence interval [CI] 0.962–0.988), African-American (HR = 3.233; 95% CI 2.131–4.906) and Texan Hispanic (HR = 2.806; 95% CI 1.556–5.601) ethnicities, and the number of ACR criteria (renal criterion excluded) (HR = 1.415; 95% CI 1.286–1.558). Male gender and hypertension were also associated with this outcome (HR = 1.647 and 1.453, respectively), but statistical significance was not reached.

**Table 3:**
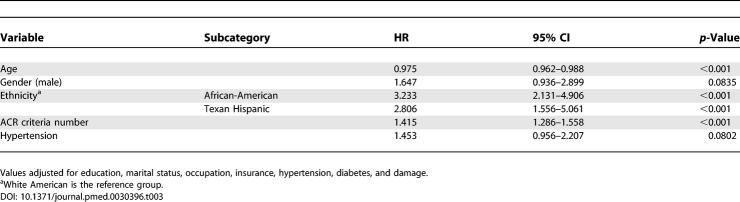
Baseline Predictors of Time to Renal Involvement in PROFILE Patients by Cox Multivariable Proportional Hazards Regression Analysis

To consider possible treatment center effects, we included both treatment center and ethnicity in alternative models of time to renal involvement. Given that all patients at UPR are from the same ethnic group, these patients were excluded in these analyses. The same variables were retained in those alternative models, therefore excluding a treatment center effect as a contributing variable to the development of renal involvement.

### Factors Associated with the Occurrence of Renal Involvement

Table 4 depicts the variables independently associated with the occurrence of renal involvement, regardless of the time when it occurred (*n =* 438). These variables were African-American ethnicity (odds ratio [OR] = 3.311; 95% CI 2.366–4.634), male gender (OR = 2.703; 95% CI 1.546–4.717), number of ACR criteria at cohort entry (OR = 1.491; 95% CI 1.321–1.684), disease duration (OR = 1.073; 95% CI 1.035–1.111), and hypertension (OR = 2.595; 95% CI 1.823–3.695). Older age (OR = 0.963; 95% CI 0.950–0.976), education (OR = 0.914; 95% CI 0.866–0.965), and smoking (OR = 0.406; 95% CI 0.259–0.636) were negatively associated with the occurrence of renal involvement.

**Table 4:**
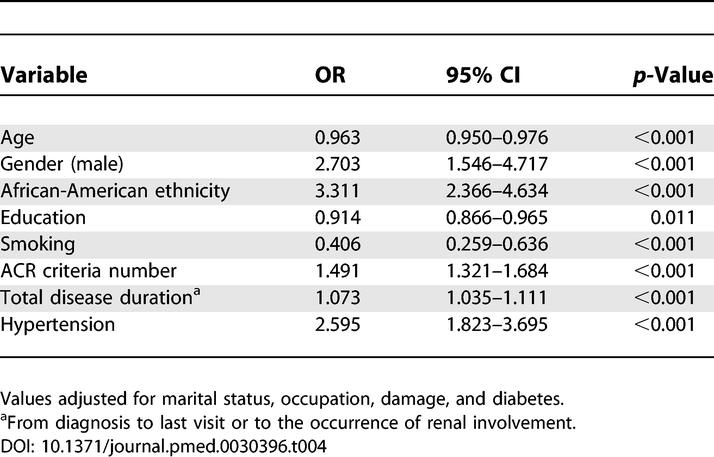
Variables Independently Associated with the Occurrence of Renal Involvement at Any Time during the Course of SLE in PROFILE Patients

### Time to ESRD

Table 5 shows the variables independently associated with time to the occurrence of ESRD among all those PROFILE patients who had developed renal involvement (prevalent and incident cases). These variables were younger age (HR = 0.958; 95% CI 0.919–0.998), Texan Hispanic ethnicity (HR = 6.771; 95% CI 1.119–40.958), and the presence of FCGR3A*GG (HR = 5.142; 95% CI 1.193–22.173). Polymorphisms of *FCGR2A* and *FCGR3B* were not retained in this model.

**Table 5:**
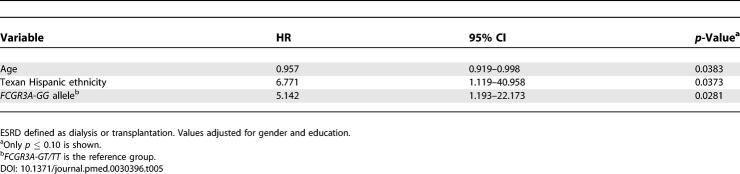
Baseline Factors Associated with Time to ESRD among 438 PROFILE Patients with Renal Involvement by Cox Multivariable Proportional Hazards Regression Analysis

## Discussion

We have previously shown that renal involvement is more frequent among SLE patients of African-American and Texan Hispanic (primarily Mexican) ancestry [28]. We have now shown that, in addition, the pace at which renal involvement occurs is also faster in these patients as compared to both White Americans and Puerto Rican Hispanics. Furthermore, we have shown, to our knowledge for the first time, that a polymorphism of FCGR3A (FCGR3A*GG) predisposes lupus patients with renal involvement to the occurrence of ESRD over and above other socioeconomic–demographic and clinical variables included in the model.

In addition to African-American and Texan Hispanic (Mexican) ancestry, younger age, hypertension, and having a greater number of ACR criteria (renal criterion excluded) were associated with a shorter time to the occurrence of renal involvement. We did find association of the FCGR3A*T allele with the lupus phenotype in White American patients in our cohort relative to a geographically and ethnically matched population (OR = 1.24, 95%CI: 1.01–1.52; *p =* 0.024). Interestingly, we did not find an association with renal involvement as opposed to non-renal lupus. Based on the meta-analyses of Karassa et al. [17] and Seligman et al. [18], this absence may represent a relative lack of statistical power for this effect, which has an estimated OR of 1.2. Similarly, the absence of an association of *HLA-DRB1, HLA-DQB1,* and *HLA-DQA1* alleles with renal involvement in our cohort may represent a power issue for HLA-DRB1*15 (HLA-DR2) and other alleles previously reported to be associated with lupus nephritis [17,29]. Given the exploratory nature of our analyses, however, a formal power calculation was not done a priori.

The finding that the FCGR3A*T allele is associated with the global lupus phenotype but that homozygosity for the FCGR3A*G allele is a risk factor for ESRD suggests that genetic factors influencing the tempo and severity of end organ disease progression may be distinct from those determining initial disease susceptibility. Indeed, work in mouse models suggests that end organ damage may be regulated differently from disease susceptibility [30]. It is likely that the low binding allele of *FCGR3A* predisposes to the global SLE phenotype by virtue of its relative lowered capacity to handle immune complexes. In contrast, once organ damage is initiated, the higher binding allele of *FCGR3A* may cause more activation and greater local damage, consistent with our observed association of ESRD with FCGR3A*GG.

Given that most variables included in the multivariable analyses were from the baseline visit, we felt that a Cox proportional hazard multivariable analysis including only patients who developed renal involvement after diagnosis (incident cases) was the most appropriate to examine our data. Nonetheless, when all patients who developed renal involvement were included in multivariable analyses, the results were consistent with the ones presented in Table 3. Interestingly, male gender and hypertension became significant, whereas smoking was negatively associated with the occurrence of renal involvement, perhaps suggesting that our smokers were overall healthier than the non-smokers.

In the analysis of time to the occurrence of ESRD, we included all patients who developed renal involvement regardless of the time at which it was first detected (incident and prevalent cases). Texan Hispanic patients (Mexican ancestry) were more likely to develop ESRD than patients in the other ethnic groups, as demonstrated in both the univariable and multivariable analyses. We not only found that FCGR3A*GG was overrepresented in those patients who went on to develop ESRD, but also that patients with this genotype were also more likely to experience a shorter time to the occurrence of ESRD (as compared to those patients with either FCGR3A*GT or FCGR3A*TT). Differences in the distribution of *FCGR3A* alleles across the ethnic groups were not the explanation for these findings, as both we and others have found the allele frequencies comparable across Hispanics, African-Americans, and White Americans (Table S2). Younger age was a significant factor in these analyses, but education was not. Nevertheless, given that ethnicity is not merely a biological construct but one that also encompasses ancestry, geography, history, values, culture, and language, the role of socioeconomic factors in the evolution to ESRD cannot be easily dismissed.

Our study has several limitations. First, it includes SLE patients recruited at academic health centers, and, thus, they may not be representative of the overall lupus population; this may make our data less generalizable than if our patients have been recruited from the wider community. Second, because different disease activity indices such as the SLEDAI (Systemic Lupus Erythematosus Disease Activity Index) [31,32] and the SLAM-R (Systemic Lupus Activity Measure–Revised) [33,34] cannot be pooled, we were unable to assess the relationship of ongoing non-clinical renal disease activity with the occurrence of renal involvement [28]. Likewise, we were unable to include histopathological and autoantibody data, such as anti-dsDNA antibodies, given that they were unavailable in a sizable proportion of patients (about 45% in the case of histopathology) or that they were not obtained at the same time in the course of the disease and were not assayed in a single laboratory using the same technique in all cohort patients (in the case of autoantibodies). Although our patients were not treated using exactly the same protocol, the proportion of patients who received individual immunosuppressants and the dose of glucocorticoids used were comparable between those whose renal disease evolved into ESRD and those whose disease did not. However, immunosuppressants as a group were more commonly used among those whose condition evolved into ESRD than those whose condition did not. Although it would have been preferable if all patients in this combined cohort had been treated using a standardized protocol, we do not think that progression to ESRD resulted primarily from differences in treatment. The roles of access to care, compliance with treatment, and family history of renal disease, while potentially important in the evolution from renal involvement to ESRD [4,35–38], were not examined in a standard manner, and thus such data were not available for pooled analyses.

Despite these limitations, our data are quite relevant to clinicians caring for patients from ethnic minorities, particularly young Hispanics (primarily of Mexican and Central American ancestry) and African-Americans, who tend to have a lower socioeconomic status in the US than the White American majority. Whether, like African-Americans, Hispanic patients of Mexican ancestry are less likely to respond to conventional treatment with pulse cyclophosphamide once renal involvement ensues deserves further examination [39]. Furthermore, our study provides insightful information as to the possible genetic basis underlying the progression to ESRD in lupus patients with renal involvement. As new treatments are developed for lupus nephritis, patients from these and other minority groups should be included to ensure that data generated in these trials are applicable to the SLE patients of various ancestral backgrounds.

## Supporting Information

Table S1 Frequency Distribution of Selected *HLA-DRB1* and *HLA-DQB1* Alleles in PROFILE Patients by Ethnic Group (46 KB DOC)

Table S2 Frequency Distribution of the *FCGR3A* Alleles in PROFILE Patients as a Function of Ethnic Group (39 KB DOC)

## Abbreviations

ACR: American College of Rheumatology
CI: confidence interval
ESRD: end-stage renal disease
HR: hazard ratio
OR: odds ratio
SDI: Systemic Lupus International Collaborating Clinics Damage Index
SLE: systemic lupus erythematosus
UPR: University of Puerto Rico Medical Sciences Campus
