# Supplementary material for: Time to Renal Disease and End-Stage Renal Disease in PROFILE: A Multiethnic Lupus Cohort
Source: PLoS Med. 2006 Oct 31;3(10):e396. doi: 10.1371/journal.pmed.0030396 (PMC1626549; doi:10.1371/journal.pmed.0030396)
Supplement: Table S1 — (46 KB DOC) [file pmed.0030396.st001.doc]

**Appendix**

**Table S1**

**Frequency Distribution of Selected *HLA-DRB1* and *HLA-DQB1* Alleles in PROFILE Patients by Ethnic Group**

| Allele | Texan Hispanic | Puerto Rican Hispanic | African American | Caucasian | All | *p* value |
| --- | --- | --- | --- | --- | --- | --- |
| *HLA-DRB1*0301* | 20.6  5.6  34.6  22.4  22.4 | 16.5  5.1  11.4  19.0  27.9 | 17.2  32.8  15.1  19.3  47.0 | 38.2  3.1  5.5  37.6  31.9 | 27.3  13.7  12.4  28.2  35.7 | <0.0001 |
| *HLA-DRB1*1503* |  |  |  |  |  | <0.0001 |
| *HLA-DRB1*08* |  |  |  |  |  | <0.0001 |
| *HLA-DQB1*0201* |  |  |  |  |  | <0.0001 |
| *HLA-DQB1*0602* |  |  |  |  |  | <0.0001 |
