# Supplementary material for: Time to Renal Disease and End-Stage Renal Disease in PROFILE: A Multiethnic Lupus Cohort
Source: PLoS Med. 2006 Oct 31;3(10):e396. doi: 10.1371/journal.pmed.0030396 (PMC1626549; doi:10.1371/journal.pmed.0030396)
Supplement: Table S2 — (39 KB DOC) [file pmed.0030396.st002.doc]

**Appendix**

**Table S2**

**Frequency Distribution of the *FCGR3A* Alleles in PROFILE Patients as a Function of Ethnic Group**

| Allele | Texan Hispanic | Puerto Rican Hispanic | African American | Caucasian |
| --- | --- | --- | --- | --- |
| *FCGR3A*GG* | 5.2  43.8  51.0 | 5.7  32.1  62.3 | 11.2  46.5  42.3 | 8.1  41.4  50.6 |
| *FCGR3A*GT* |
| *FCGR3A*TT* |

*p=0.097
